# Broad range detection of viral and bacterial pathogens in bronchoalveolar lavage fluid of children to identify the cause of lower respiratory tract infections

**DOI:** 10.1186/s12879-021-05834-0

**Published:** 2021-02-05

**Authors:** Heping Wang, Jiali Gu, Xiaonan Li, Christa E. van der Gaast-de Jongh, Wenjian Wang, Xuehui He, Zhi Xu, Yonghong Yang, Ronald de Groot, Marien I. de Jonge, Yuejie Zheng

**Affiliations:** 1 Shenzhen Children’s Hospital, No. 7019 Yitian Road, Futian District, Shenzhen, 518038 Guangdong China; 2 Section Pediatric Infectious Diseases, Laboratory of Medical Immunology, Radboud Center for Infectious Diseases, Radboud university medical center, Philips van Leydenlaan 15, 6525 EX Nijmegen, The Netherlands; 3 Ningbo Health Gene Technologies Co., Ltd., Ningbo, Zhejiang China

**Keywords:** Children, Bronchoalveolar lavage fluids, Pathogen detection, Lower respiratory tract infections

## Abstract

**Background:**

Knowledge on the etiology of LRTIs is essential for improvement of the clinical diagnosis and accurate treatment. Molecular detection methods were applied to identify a broad range of bacterial and viral pathogens in a large set of bronchial alveolar lavage (BAL) fluid samples. The patterns of detected pathogens were correlated to the clinical symptoms.

**Methods:**

BAL fluid samples and clinical data were collected from 573 hospitalized children between 1 month and 14 years of age with LRTIs, enrolled from January to December 2018. Pathogens were detected using standardized clinical diagnostics, with a sensitive, high-throughput GeXP-based multiplex PCR and with multiplex qPCR. Data were analyzed to describe the correlation between the severity of respiratory tract disease and the pathogens identified.

**Results:**

The pathogen detection rate with GeXP-based PCR and multiplex qPCR was significantly higher than by clinical routine diagnostics (76.09% VS 36.13%,χ^2^ = 8.191, *P* = 0.004). The most frequently detected pathogens in the BAL fluid were human adenovirus (HADV)(21.82%), *Mycoplasma pneumoniae* (20.24%), human rhinovirus (13.96%), *Streptococcus pneumoniae* (8.90%) and *Haemophilus influenzae* (8.90%). In 16.4% of the cases co-detection with two or three different pathogens was found. Viral detection rates declined with age, while atypical pathogen detection rates increased with age. Oxygen supply in the HADV and Influenza H1N1 infected patients was more frequent (49.43%) than in patients infected with other pathogens.

**Conclusion:**

Broad range detection of viral and bacterial pathogens using molecular methods is a promising and implementable approach to improve clinical diagnosis and accurate treatment of LRTI in children.

**Supplementary Information:**

The online version contains supplementary material available at 10.1186/s12879-021-05834-0.

## Background

Lower respiratory tract infections (LRTI) are the leading cause of morbidity and mortality in children aged < 5 years worldwide [1]. Of the estimated 5.9 million deaths in children younger than 5 years, pneumonia was found to be the most important cause of death (16%) [2]. The etiology is complex as a whole range of different pathogens, mainly viruses and bacteria, in different combinations, can cause LRTI. Accurate detection of respiratory tract pathogens is crucial for the clinical diagnosis and for the epidemiology of LRTIs [3].

Bronchoalveolar lavage (BAL) has been considered the gold standard for detection of the causative agents of lower respiratory tract infections. However, BAL is only performed in patients with complex or severe airway diseases, e.g. children with chronic lung diseases, such as cystic fibrosis, or recurrent LRTIs [4–6]. However, in the literature, studies with a small number of inclusions [7, 8] or studies focused on a limited number of pathogens can be found, focusing either only on viruses [4, 9] or on bacterial pathogens [10]. However, large studies detecting both viral and bacterial pathogens are needed to better understand the cause-effect relation, the course of disease and the level of severity to finally improve treatment and accelerate the recovery from LRTI.

The utilization of molecular detection methods will reduce the turnaround time for diagnosis and will enable the detection of more than one pathogen simultaneously. In this study, we detected a total of 21 different pathogens: 11 viruses and 2 bacterial pathogens by GeXP-PCR [11] and 8 bacterial pathogens by 2 in-house qPCR assays [12] in BAL fluids from children admitted to Shenzhen Children’s Hospital. The results on the molecular detection of the pathogens in the BAL fluid were combined with the clinical characteristics of the patients with LRTI rendering more insight into the causative agents and diseases severity. Furthermore, this study provides valuable data to improve treatment regimens and to guide vaccine development strategies in China.

## Materials and methods

### Samples collection and information

Patients with complex or severe airway disease were enrolled in this study from January to December 2018. Patients eligible for BAL sampling were those with a persistent chest radiographic infiltrate and/or persistent respiratory symptoms, such as cough, wheezing, dyspnea, exacerbation, or unexplained hypoxemia. The collection of BAL fluid was done within the Department of Respiratory Diseases in Shenzhen Children’s Hospital for pathogen identification using standardized clinical diagnostic assays, as described here below, to facilitate the clinical decision for treatment. Furthermore, the same samples were analyzed with two different PCR methods (one for the detection of viral and atypical pathogens and one for the detection of bacterial pathogens), as described here below. Clinical and demographic data of the patients enrolled in this study were retrieved from the Shenzhen Children’s Hospital electronic patient dossiers. This study was approved by the Ethical Committee of Shenzhen Children’s Hospital with registration number 2016013. Written informed consent was obtained from the parents and caregivers of included children before study enrollment.

### Standardized clinical diagnostics on BAL fluids

BAL fluids were collected from the infected part of children with infection and/or the large airways of children with persistent respiratory symptoms and sent to the clinical lab for standardized diagnostic analyses within 2 h after sampling. Total DNA was extracted from 1 mL of BAL fluids, *Mycoplasma pneumoniae* was diagnosed using the TaqMan probe PCR kit for *M. pneumoniae* (DaAn Gene Co. Ltd., Guangzhou, China). We utilized 3 mL of BAL fluids to detect viruses, the D3 Ultra Direct Immunofluorescence Assay (DFA) Respiratory Virus Screening and ID Kit (Diagnostic hybrids, Inc., Athens, Ohio, USA) was employed to detect respiratory syncytial virus, adenovirus, influenza A/B virus and parainfluenza virus (types 1, 2 and 3). We utilized 3 mL of BAL fluids for bacterial culture. Bacterial culturing was conducted to detect *Acinetobacter baumannii*, *Haemophilus influenzae*, *Haemophilus parainfluenzae*, *Moraxella catarrhalis*, *Staphylococcus aureus*, *Staphylococcus haemolyticus* and *Streptococcus pneumoniae.*

### GeXP-based multiplex PCR and in-house qPCR on BAL fluid samples

Total RNA/DNA was extracted from 300 μL respiratory tract secretion using the TAN Bead Opti Pure Viral Auto Tube (Taiwan Advanced Nanotech, Taiwan, China) on Smart LabAssist-32 (Taiwan Advanced Nanotech, Taiwan, China) according to the manufacturer’s instruction. The extracts were eluted into 80 μL of DNase- and Rnase-free water and then quantified using spectrophotometry. Plasmid pcDNA3.1 (+) (Thermo Fisher Scientific Co Ltd., Shanghai, China) was used as internal control. The GeXP-based assay (Genome Lab GeXP Genetic Analysis System) was performed on all BAL fluid samples for 13 different respiratory pathogens: human adenovirus (HADV), *Chlamydophila pneumoniae*, coronaviruses (229E, OC43, NL63 and HKU1), human metapneumovirus (HMPV), human rhinovirus (HRV), human bocavirus (Boca), influenza A (H3N2, H1N1, H5N2 and H7N9) and B viruses, *Mycoplasma pneumoniae*, parainfluenza virus (types 1, 2, 3 and 4) and human respiratory syncytial virus (HRSV), using the Respiratory Pathogens Multiplex Kit (PCR-Capillary Electrophoresis Fragment Analysis) (Health Gene Tech., Ningbo, China). The data was analyzed with the Ge-XP system Software [11, 13]. In addition, we simultaneously performed semi-quantitative PCR detection on adenovirus-positive specimens using the TaqMan probe PCR kit for HADV (Shenzhen Puruikang Biotechnology Co., Ltd., Shenzhen, China).

Bacterial loads were assessed by an in-house qPCR assay for *S. pneumoniae, H. influenzae, M. catarrhalis, S. aureus, E. coli, K. pneumoniae, P. aeruginosa, and A. baumannii* and were expressed as colony forming unit (CFU) equivalents per milliliter using standard curves generated by target gene plasmid dilution series as previously described [12].

### Statistical analysis

Statistical analyses were conducted using SPSS 19 (SPSS Inc. Chicago, IL, USA). For comparison of categorical data, chi-square or Fisher’s exact test was used.

## Results

### Patient characteristics

The median age of the 573 enrolled pediatric patients requiring a BAL was 28 months (interquartile range, 12 to 60), and the median length of hospital stay was 8 days (interquartile range, 6 to 10). Male to female ratio of the patients was 1.65:1. The majority of patients (82.6%) had a radiologically confirmed pneumonia and persistent respiratory symptoms (17.5%). A total of 192 patients (33.5%) required oxygen supply and 5 (0.9%) required invasive mechanical ventilation (Table 1). None of the patients were admitted to the ICU.

**Table 1:** Clinical and demographic characteristics of the patients included in this study

Characteristics	Children requiring bronchoalveolar lavage (Total number = 573) (%)
Age (months)	
< 12	168 (29.3)
12–36	162 (28.3)
37–72	132 (23.0)
73–168	111 (19.4)
Gender	
Male	357 (62.3)
Female	216 (37.7)
Any underlying conditions	
Asthma or allergic diseases	110 (19.2)
Congenital diseases	43 (7.5)
Symptom	
Fever	370 (64.6)
Cough	506 (88.3)
Wheezing	99 (17.3)
Diagnosis based on Chest X-ray or CT scan	
Pneumonia	442 (77.1)
Atelectasis (partial or complete)	116 (20.2)
Pleural effusion	65 (11.3)
Bronchiectasis	56 (9.8)
Consolidation	55 (9.6)
Hospitalization	
Median length of stay	8 days
Mechanical ventilation	5 (0.9)
Oxygen absorption	192 (33.5)
Antibiotic treatment	524 (91.5)
Antiviral treatment	76 (13.3)
Time of bronchoscopy	
Median time post-admission	4 days

The 573 patients were divided in four age groups: infants (1–12 months, 168 cases), toddlers (~ 3 years old, 162 cases), preschool children (~ 6 years old, 132 cases) and school children (~ 14 years old, 111 cases). No significant differences in the prevalence of LRTI were found between the age groups (Table 1).

### Pathogen detection using standardized clinical diagnostic tests

In 207 out of 573 samples (36.1%) one or more pathogens were detected. In 87 samples (15.2%) 2 or more pathogens were detected, strongly suggesting that these patients had a co-detection. The most common pathogens detected by routine clinical laboratory diagnostics were *M. pneumoniae* (in 20.2% of the patients), by a clinically validated PCR, *S. pneumoniae* (5.76%) and *H. influenzae* (3.1%) by culture, and human adenovirus (3%) by DFA (Supplementary Table 1).

### Pathogen detection by GeXP-based multiplex PCR and qPCR

**Fig. 1:**
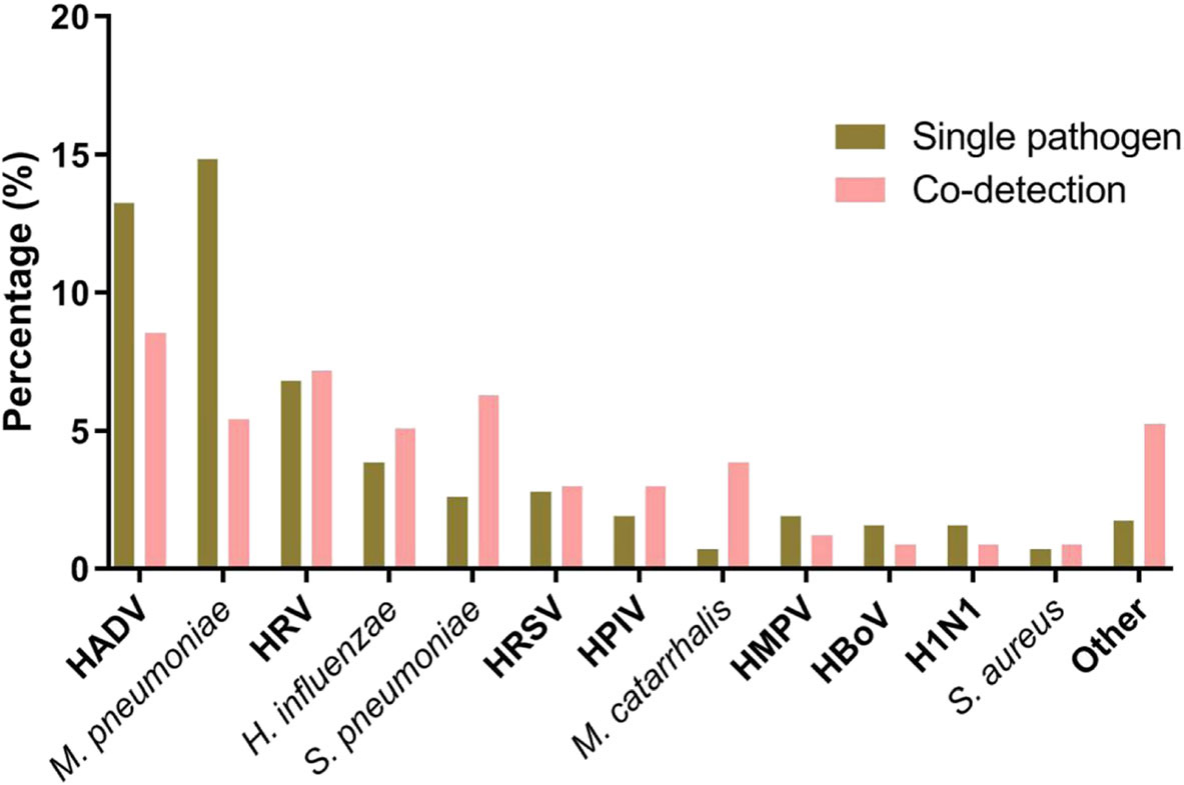
Percentages of viral and bacterial pathogens detected in 573 BAL fluids, including single- and co-detection, ‘other’ includes HCoV, INFB, *C. pneumoniae, Klebsiella pneumoniae, Pseudomonas aeruginosa, Escherichia coli,* and *Acinetobacter baumannii*

A total of 436 BALFs (76.1%) were positive for at least one pathogen, of which 19.9% had a co-detection. The most frequently detected pathogens using these PCR-based methods were HADV (21.8%), *M. pneumoniae* (20.2%), HRV (14%), *S. pneumoniae* (8.9%) and *H. influenzae* (8.9%), followed by HRSV (5.8%), HPIV (4.9%), *M. catarrhalis* (4.5%), HMPV (3.1%), Boca (2.4%), H1N1 (2.4%), *S. aureus* (1.6%) and HCOV (1.2%) (Fig. 1).

**Fig. 2:**
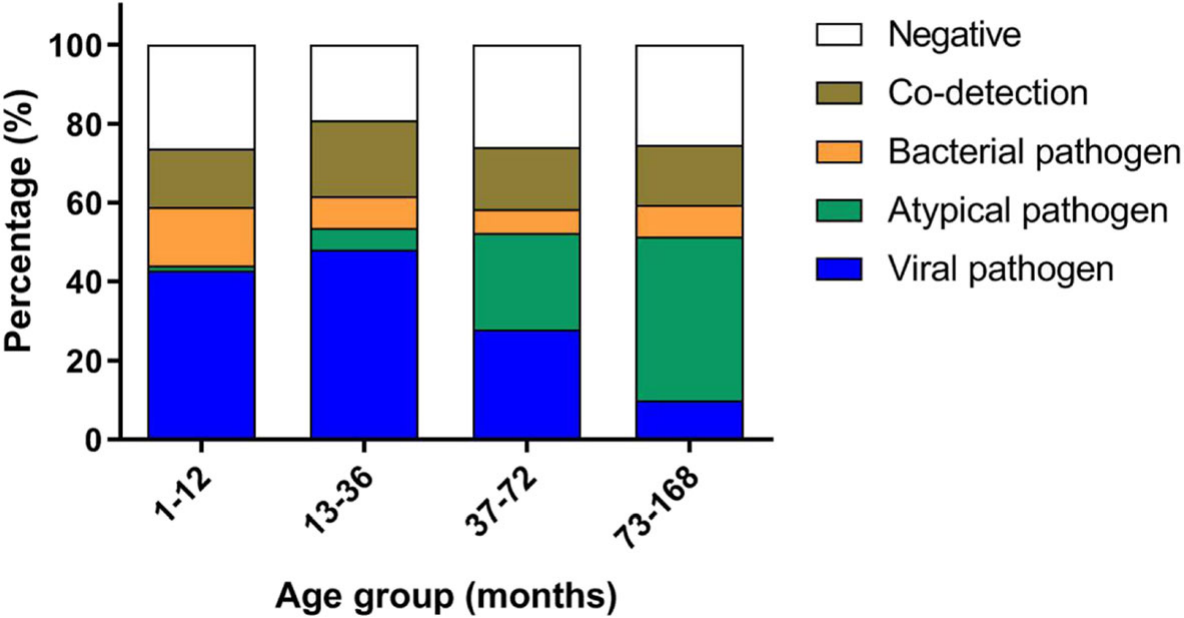
Detection rates of the different pathogen groups (viral, bacterial, atypical, co-detection, no pathogen) based on age. The atypical pathogens include *M. pneumoniae* and *C. pneumoniae*

A total of 198 patients (34.6%) were infected with viruses only, while 55 (9.6%) were infected with bacterial pathogens. Co-detection with 2 or 3 different pathogens was found in 94 patients (16.4%)(Fig. 2).

**Fig. 3:**
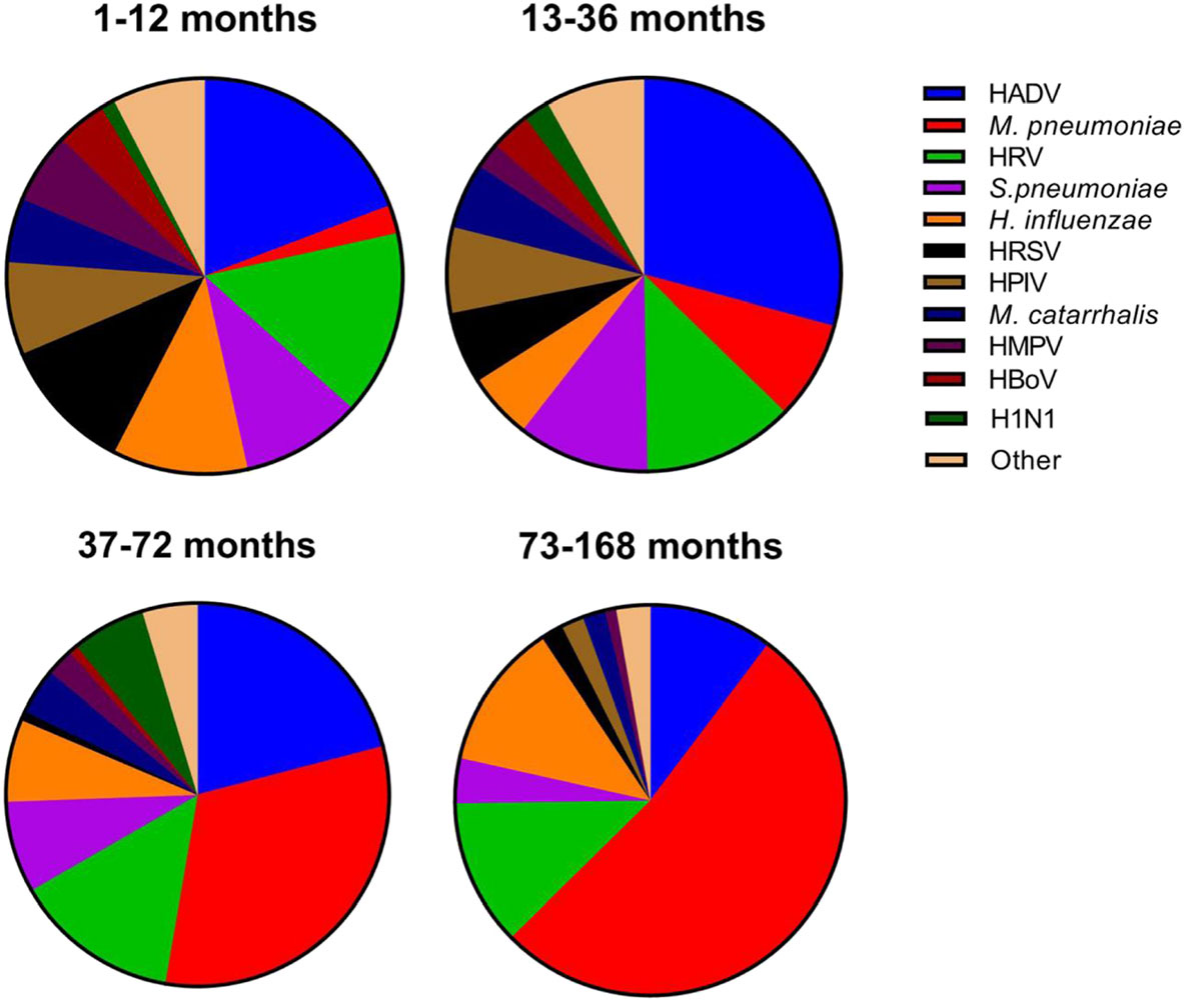
Age-dependent infection rates with specific pathogens detected in BAL fluids. ‘other’ includes HCoV, INFB, *Chlamydophila pneumoniae, Staphylococcus aureus, Klebsiella pneumoniae, Pseudomonas aeruginosa, Escherichia coli,* and *Acinetobacter baumannii*

Although this study included more male patients (62.3%) than female patients (37.7%), infection rates were comparable males (76.2%) and females (75.9%) (χ^2^ = 0.005, *p* = 0.943). Detection rates in different age groups were not significantly differently: 1–12 months: 73.81%, 1–3 years: 80.86%, 4–7 years: 74.24% and 8–14 years: 74.77% (χ^2^ = 2.863, *p* = 0.413). In general, viral lower respiratory tract infection rates declined with age, while infection with atypical pathogen rates increased with age. Bacterial infection of the lungs peaked during infancy. HADV and *S. pneumoniae* were detected more commonly in children younger than 6 years old, while *M. pneumoniae* detection rate increased with age significantly (Fig. 3).

**Fig. 4:**
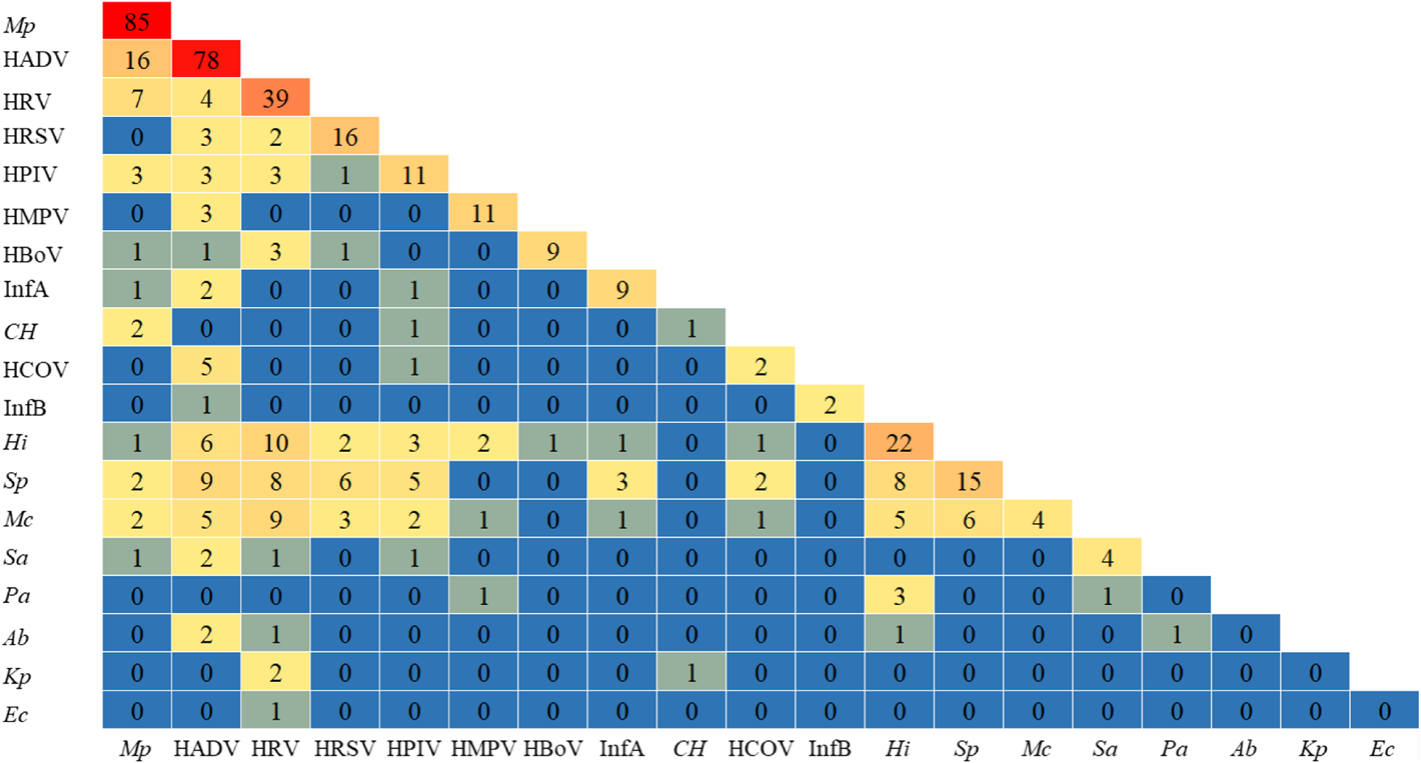
Specific combinations of pathogens identified in BAL fluid from pediatric patients with lower respiratory tract infections. Abbreviations in the figure: *Mycoplasma pneumoniae* (*Mp*), *Chlamydia* (*CH*), *Haemophilus influenzae* (*Hi*), *Strepcoccus pnemoniae (Sp), Moraxella catarrhalis (Mc), Staphylococcus aureus* (*Sa*)*, Klebsiella pneumoniae* (*Kp*)*, Pseudomonas aeruginosa* (*Pa*)*, Escherichia coli* (*Ec*)*,* and *Acinetobacter baumannii* (*Ab*)

Of all pathogens identified, *M. catarrhalis* showed the highest co-detection rate (84.6%). Bacterial co-detection rates were higher than viral co-detection rates (Fig. 4).

### Comparison of pathogen detection in children with and without pneumonia

**Fig. 5:**
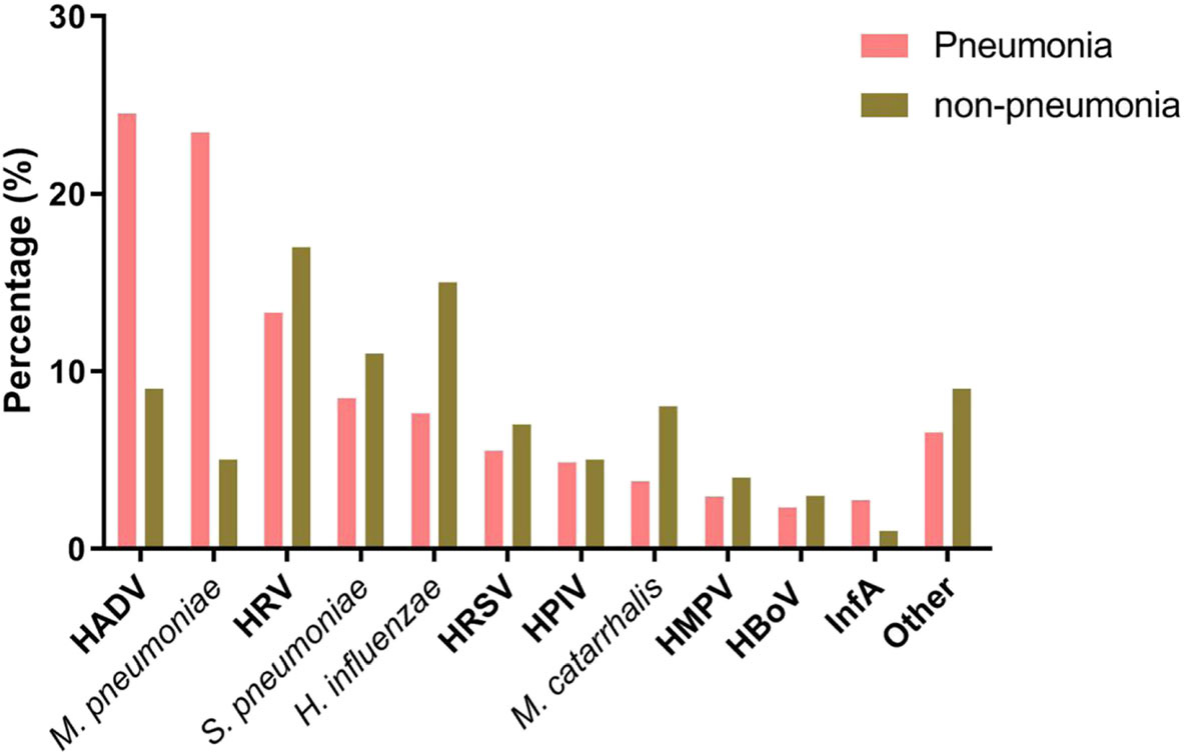
Specific pathogens were detected in BAL fluids between pneumonia group and non-pneumonia group. ‘other’ includes HCoV, INFB, *Chlamydophila pneumoniae, Staphylococcus aureus, Klebsiella pneumoniae, Pseudomonas aeruginosa, Escherichia coli,* and *Acinetobacter baumannii*

The detection rate of pathogens in children with pneumonia (78.4%, 371/473) was significantly higher than in children without pneumonia (65.0%, 65/100, χ^2^ = 8.191, *P* = 0.004). The majority of atypical pathogens were detected in children with pneumonia, while more bacterial pathogens were detected in the non-pneumonia than in the pneumonia group (Fig. 5). HADV and *M. pneumoniae* were more frequently detected in BAL samples from children with pneumonia, *H. influenzae* was more often detected in children without pneumonia (Fig. 5).

### The causative agents were missed by routine laboratory tests in children requiring BAL

The overall detection rate of pathogens by GeXP-based assay and multiplex qPCR (76.1%) was significantly higher than that by clinical laboratory tests (36.1%). In addition, the GeXP-based PCR has a much broader detection range and included *Chlamydophila pneumoniae* human rhinovirus (HRV), HMPV, HCOV and Boca virus. The sensitivity of GeXP-based assay was similar to single qPCR. The detection rates of GeXP-based assay were significantly higher than with DFA; HADV (21.8% vs 3%), HRSV (5.8% vs 2.6%), HPIV (4.9% vs 0.9%)and INFA/B (3% vs 0.52%). In those cases that HADV was detected as a single pathogen the Ct values were significantly lower than when co-detected with other pathogens, median values of HADV as single pathogen and when co-detected with other pathogens were Ct 20.35 and Ct 29.45, respectively (supplementary Table 2A and 2B). The DFA was more likely to be positive when the Ct values were lower. Bacterial detection rates by qPCR were higher than culture, but there was no significant difference between these two assays (Supplementary Table 1).

### The correlation between single pathogen detected in the BAL fluids and clinical signs

We divided the single pathogen-infected children into 3 groups according to pathogen (atypical, viral and bacterial pathogens) and analyzed the relation between respiratory diseases and pathogens, *M. pneumoniae* (85 cases), virus (171 cases), *H. influenzae* or *S. pneumoniae* (37 cases). As demonstrated in Table 2, fever, oxygen supply, WBC (white blood cell), Neutrophil counts, CRP (C-reactive protein), PCT (procalcitonin) were statistically different among groups of bacteria, mycoplasma and virus. The fever rate of patient infected with *M. pneumoniae* was significantly higher than bacterial or viral infection. More patients infected with virus needed oxygen supply.

**Table 2:** Clinical symptoms and treatments. Data are median (IQR), n (%), or n/N (%). The *p* values were calculated by Kruskal-Wallis H test, χ^2^ test, or Fisher’s exact test, as appropriate

	Bacterial infection (non-***M. pneumoniae***)	***M. pneumoniae*** infection	Viral infection	***p*** value
**Fever**				
**0**	22 (59.5)	6 (7.1)	49 (28.7)	< 0.001
**1**	15 (40.5)	79 (93)	122 (71.4)	
**Cough**				
**0**	5 (13.5)	3 (3.5)	9 (5.3)	0.0854
**1**	32 (86.5)	82 (96.5)	162 (94.7)	
**Oxygen supply**				
**0**	33 (89.19)	64 (75.29)	95 (55.6)	< 0.001
**1**	4 (10.81)	21 (24.71)	76 (44.4)	
**Antibiotics**				
**0**	1 (2.7)	-(−)	2 (1.2)	0.3783
**1**	36 (97.3)	85 (100)	169 (98.8)	
**Antiviral treatment**				
**0**	33 (89.2)	77 (90.6)	139 (81.3)	0.1088
**1**	4 (10.8)	8 (9.4)	32 (18.7)	
**Steriod treatment**				
**0**	20 (54.1)	39 (45.9)	103 (60.2)	0.0927
**1**	17 (45.9)	46 (54.1)	68 (39.8)	
**Neutrophils**				
**0**	14 (37.8)	39 (45.9)	59 (34.5)	0.2105
**1**	23 (62.2)	46 (54.1)	112 (65.5)	
**Hospitalisation days**	7 (6.0–9.0)	8 (7.0–10.0)	8 (6.0–11.0)	0.0976
**WBC (× 10**^**9**^**/L)**	10.98 (9.08–14.42)	6.58 (5.5–8.6)	9.58 (6.96–14.1)	< 0.001
**CRP (mg/L)**	3.8 (0.5–12.8)	14.9 (5.4–34)	4.5 (0.1–16.1)	< 0.001
**PCT (μg/L)**	0.2 (0.1–1.0)	0.19 (0.1–0.3)	0.17 (0.1–0.9)	0.001

## Discussion

Due to the invasive nature of bronchoscopy, data on the presence of pathogens in the lower airways are very scarce. A unique set of 573 BAL fluid samples was collected in children between January and December in 2018, in Shenzhen, Southern China. This allowed us for the first time to study the presence of pathogens in the lower respiratory tract on a relatively large scale. Furthermore, this study has enabled us to compare the routine clinical diagnostics with a new PCR-based diagnostics approach.

PCR-based diagnostics resulted in detection of respiratory pathogens in 76.1% of all cases. A similar percentage (78.7%) as was previously found in a smaller study recently conducted in Zhengzhou (Northern China) [10], but higher than previously found in studies performed in Suzhou and Shanghai (Eastern China) [3, 14]. While the routine clinical diagnostics approach in this study resulted in the detection of respiratory pathogens in 36.1%. Which clearly indicates that PCR-based detection is far more sensitive than the routine clinical diagnostics as currently applied. PCR-based detection also revealed that co-detection was found in 16.4%. Most prevalent of all co-detections were viral-bacterial co-detections (11.2%). The co-detection rate was lower than that in Zhengzhou (30.6%) and similar to that in Shanghai (11.4%), also determined by PCR-based detection.

Interestingly, HADV and *M. pneumoniae* showed similar clinical characteristics, including the fact that 90% of patients infected with these pathogens developed fever. However, the median age of children infected with HADV, was significantly younger than children infected with *M. pneumoniae.* Strikingly, this study confirms that HADV is a common pathogen in Southern China (Guangzhou, Hunan) as high prevalence was earlier found in other studies conducted in the same region [15, 16]. This was not the case for other areas in China, which is concluded based on studies conducted in Suzhou and Chongqi [17, 18]. However, *M. pneumoniae* seems highly prevalent in different parts of China. This striking regional difference within China demands for further study.

HRV was detected in 13.96% of the patients and was the most common pathogen in patients with severe pneumonia. HRV was more often co-detected with other pathogens than found as single pathogen.

*H. influenzae* and *S. pneumoniae* were the most common bacterial causes of LRTIs. *H. influenzae* is often found in patients with chronic cough and protracted bacterial bronchitis (PBB) [19]. We found *H. influenzae* was more detected in children without pneumonia in this study, this might be due to the selection of patients and the number of cases. The qPCR method for detection of bacterial pathogens was more sensitive than bacterial culture.

Strikingly not RSV but HADV was the most important viral pathogen in this study as opposed to previous studies [15, 20]. This could be explained by, method of detection, study population and geographical location.

This study has some limitations. Firstly, none of the diagnostic methods was able to detect fungal pathogens, while fungi might be important pathogens of LRTIs in part of the patients. Secondly, we cannot exclude that we might have missed some of the RNA viruses because of RNA degradation during storage and sample processing. Thirdly, the amount of fluid retrieved and sampling conditions could have influenced the detection rate. Fourth, in 10.3% of the children the BAL sample was taken more than 1 week after admission because of different reasons. A combination of the above mention factors could possibly explain why in 23,9% of the cases no viral or bacterial pathogen was found. Nevertheless, the molecular methods used in this study significantly increased the diagnostic accuracy (from 36.1 to 76.1%).

In conclusion, lower respiratory tract infections are complex with a diverse clinical phenotype and are caused by many different pathogens. We found that viruses were the major pathogens detected in the BAL fluids. Viral and bacterial pathogens were detected more frequently in younger children. This study gave us insight into the etiology of LRTI in children from different ages from Southern China and will help to improve the diagnosis. GeXP-based multiplex PCR with a much broader range of pathogen detection clearly showed to be superior than the routine laboratory diagnostic methods, showing promise for the near future of LRTI diagnostics.

## Conclusions

HADV, rhinovirus and *Mycoplasma pneumoniae* are the most important causes of severe LRTI in children in the south of China. Viral detection rates declined with age, while atypical pathogen detection rates increased with age. These promising molecular methods of pathogen detection will be implemented to improve clinical diagnosis and accurate treatment of LRTI in children.

## Supplementary Information

**Additional file 1 Table S1**. Pathogens detected by CD or PCR.

**Additional file 2 Table S2**. Ct values of human adenovirus (HADV) detected in the BAL fluid.

## Data Availability

The key information and data generated and/or analyzed during this study were included in this article and/or its supplementary information files.

## Abbreviations

LRTIs: Lower respiratory tract infections
BAL: Bronchoalveolar lavage
PCR: Polymerase chain reaction
HADV: Human adenovirus
DFA: Direct immunofluorescence assay
HMPV: Human metapneumovirus
HRV: Human rhinovirus
Boca: Human bocavirus
HRSV: Human respiratory syncytial virus
HCOV: Human coronavirus
HPIV: Human parainfluenza virus
CFU: Colony forming unit
WBC: White blood cell
CRP: C-reactive protein
PCT: Procalcitonin
