# Supplementary material for: Broad range detection of viral and bacterial pathogens in bronchoalveolar lavage fluid of children to identify the cause of lower respiratory tract infections
Source: BMC Infect Dis. 2021 Feb 5;21:152. doi: 10.1186/s12879-021-05834-0 (PMC7864134; doi:10.1186/s12879-021-05834-0)
Supplement: Supplementary file 2 — Additional file 2 Table S2. Ct values of human adenovirus (HADV) detected in the BAL fluid. [file 12879_2021_5834_MOESM2_ESM.docx]

**Supplementary Table 2**

1. **HADV only:**

| **Patient nr.** | **Ct value** | **DFA+/-** |
| --- | --- | --- |
| 3 | 16.4 | + |
| 5 | 29.4 | - |
| 13 | 16.2 | - |
| 41 | 30.0 | - |
| 42 | 14.5 | + |
| 51 | 35.0 | - |
| 53 | 29.2 | - |
| 62 | 19.3 | - |
| 71 | 17.2 | - |
| 72 | 37.4 | - |
| 73 | 39.2 | - |
| 77 | 18.5 | + |
| 84 | 18.0 | + |
| 109 | 20.6 | - |
| 110 | 18.4 | - |
| 115 | 19.3 | - |
| 117 | 17.1 | - |
| 119 | 15.2 | + |
| 120 | 20.1 | - |
| 121 | 28.3 | - |
| 123 | 13.5 | + |
| 125 | 33.5 | - |
| 126 | 13.4 | + |
| 127 | 16.0 | - |
| 132 | 15.4 | - |
| 136 | 23.0 | - |
| 137 | 30.0 | - |
| 138 | 20.0 | + |
| 139 | 30.7 | - |
| 150 | 15.4 | + |
| 157 | 19.0 | - |
| 159 | 16.3 | + |
| 162 | 15.8 | + |
| 174 | 17.4 | - |
| 180 | 19.2 | - |
| 191 | 12.4 | + |
| 196 | 29.4 | - |
| 205 | 31.4 | - |
| 209 | 18.2 | - |
| 218 | 11.3 | - |
| 250 | 29.5 | - |
| 259 | 32.3 | - |
| 261 | 17.3 | - |
| 262 | 25.8 | - |
| 266 | 14.4 | - |
| 273 | 18.5 | - |
| 275 | 27.4 | + |
| 276 | 17.3 | - |
| 282 | 17.2 | - |
| 287 | 22.3 | - |
| 315 | 33.5 | - |
| 327 | 32.8 | - |
| 331 | 30.8 | - |
| 348 | 31.2 | - |
| 364 | 35.9 | - |
| 368 | 22.0 | - |
| 369 | 20.0 | - |
| 373 | 21.8 | - |
| 381 | 27.4 | - |
| 382 | 17.2 | + |
| 383 | 31.5 | - |
| 384 | 31.5 | - |
| 387 | 23.9 | - |
| 409 | 13.9 | - |
| 413 | 15.2 | - |
| 414 | 32.7 | - |
| 440 | 19.3 | - |
| 473 | 27.2 | - |
| 476 | 21.5 | - |
| 484 | 16.9 | - |
| 517 | 13.1 | - |
| 529 | 18.2 | + |
| 532 | 31.7 | - |
| 549 | 31.6 | - |
| 551 | 20.1 | - |
| 552 | 22.2 | - |
| 558 | 33.6 | - |
| 560 | 21.5 | - |
| 571 | 20.9 | - |
| 572 | 25.7 | - |

*Median Ct value: 20.35*

1. **HADV co-detection:**

| **Patient No.** | **Ct value** | **DFA+/-** |
| --- | --- | --- |
| 18 | 14.1 | + |
| 88 | 31.2 | - |
| 122 | 32.0 | - |
| 144 | 24.0 | - |
| 155 | 23.2 | - |
| 177 | 20.5 | - |
| 203 | 25.6 | - |
| 219 | 27.4 | - |
| 225 | 27.8 | - |
| 241 | 20.5 | - |
| 258 | 15.3 | - |
| 267 | 35.1 | - |
| 291 | 35.4 | - |
| 314 | 15.1 | + |
| 323 | 15.6 | + |
| 325 | 32.5 | - |
| 329 | 37.7 | - |
| 330 | 34.2 | - |
| 333 | 36.2 | - |
| 341 | 29.3 | - |
| 353 | 32.5 | - |
| 370 | 33.8 | - |
| 412 | 32.0 | - |
| 444 | 34.2 | - |
| 514 | 14.8 | + |
| 519 | 28.6 | - |
| 527 | 29.6 | - |
| 528 | 35.2 | - |
| 531 | 35.5 | - |
| 548 | 21.9 | + |
| 554 | 21.6 | - |
| 557 | 29.8 | - |
| 559 | 33.7 | - |
| 573 | 27.1 | - |

*Median Ct value: 29.45*

**Supplementary table 2.** Ct values of all patients in which human adenovirus (HADV) was detected in the BAL fluid, either as single pathogen (2A) or in combination with other pathogens (2B). Furthermore, it is indicated in which patients the Direct Immunofluorescence Assay (DFA) detecting HADV was positive (+) or negative (-).
